# Inducible nitric oxide synthase (iNOS) expression in monocytes during acute Dengue Fever in patients and during *in vitro *infection

**DOI:** 10.1186/1471-2334-5-64

**Published:** 2005-08-18

**Authors:** Patrícia CF Neves-Souza, Elzinandes L Azeredo, Sonia MO Zagne, Rogério Valls-de-Souza, Sonia RNI Reis, Denise IS Cerqueira, Rita MR Nogueira, Claire F Kubelka

**Affiliations:** 1 Departmento de Virologia, Instituto Oswaldo Cruz Fundação Oswaldo Cruz, Av. Brasil, 4365, CEP 21040-360 Rio de Janeiro, RJ, Brazil; 2 Departamento de Clínica Médica, Hospital Antonio Pedro, Universidade Federal Fluminense, Niterói, RJ, Brazil; 3 Instituto de Pesquisas Evandro Chagas, Fundação Oswaldo Cruz, Av. Brasil, 4365, CEP 21040-360 Rio de Janeiro, RJ, Brazil

## Abstract

**Abstract:**

Mononuclear phagocytes are considered to be main targets for Dengue Virus (DENV) replication. These cells are activated after infection, producing proinflammatory mediators, including tumour-necrosis factor-α, which has also been detected *in vivo*. Nitric oxide (NO), usually produced by activated mononuclear phagocytes, has antimicrobial and antiviral activities.

**Methods:**

The expression of DENV antigens and inducible nitric oxide synthase (iNOS) in human blood isolated monocytes were analysed by flow cytometry using cells either from patients with acute Dengue Fever or after DENV-1 *in vitro *infection. DENV-1 susceptibility to iNOS inhibition and NO production was investigated using N^G^-methyl L-Arginine (N^G^MLA) as an iNOS inhibitor, which was added to DENV-1 infected human monocytes, and sodium nitroprussiate (SNP), a NO donor, added to infected C6/36 mosquito cell clone. Viral antigens after treatments were detected by flow cytometry analysis.

**Results:**

INOS expression in activated monocytes was observed in 10 out of 21 patients with Dengue Fever and was absent in cells from ten healthy individuals. DENV antigens detected in 25 out of 35 patients, were observed early during *in vitro *infection (3 days), significantly diminished with time, indicating that virus replicated, however monocytes controlled the infection. On the other hand, the iNOS expression was detected at increasing frequency in *in vitro *infected monocytes from three to six days, exhibiting an inverse relationship to DENV antigen expression. We demonstrated that the detection of the DENV-1 antigen was enhanced during monocyte treatment with N^G^MLA. In the mosquito cell line C6/36, virus detection was significantly reduced in the presence of SNP, when compared to that of untreated cells.

**Conclusion:**

This study is the first to reveal the activation of DENV infected monocytes based on induction of iNOS both *in vivo *and *in vitro*, as well as the susceptibility of DENV-1 to a NO production.

## Background

Dengue viruses (DENV) have been detected in several lymphoid organs originating from fatal cases of hemorrhagic disease, mainly in cells from the mononuclear phagocyte lineage [1]. Nevertheless, the frequency of disseminated virus detection in autopsies is low and microscopic injury is not sufficient to justify death during the Dengue Hemorrhagic Fever (DHF) or Dengue Shock Syndrome (DSS). It is believed that disease severity is a result of an immunopathological response leading to hemorrhagic and vasodynamic alterations and shock.

Mononuclear phagocytes have been considered as main targets for DENV replication [2,3] and recently the virus was detected in dendritic cells originating from one individual 24 hours post DENV infection [4,5]. Morens *et al*. [6] have shown strain differences of DENV, originating either from mild or severe cases, with respect to their growth in human monocytes, suggesting that characteristics inherent to virus genome and replication were related with disease severity. Viral RNA was detected by RT-PCR in peripheral blood leukocytes [7] in humans during infection and DENV antigens were present in circulating monocytes [8], Kupffer cells and macrophages from different organs such as liver, spleen and lungs [9].

Pathogenesis may consist of virus penetration into monocytes or dendritic cells, cell activation and synthesis induction of cytokines, arachdonic acids and very likely nitric oxide. These factors would be involved not only in generating dengue disease [10] but also in the elimination of viruses [11].

The *in vivo *activation of mononuclear phagocytes by interferon (IFN)-γ during DENV infection can be expected, since this cytokine is detected in serum during the acute phase [12,13] and is also produced by peripheral leukocytes from patients with previous dengue records [14]

The *in vitro *production of IFN-α,β, interleukin-6 and tumour-necrosis factor (TNF)-α by infected fibroblasts and/or macrophages was described [15] as well as the production of platelet activating factors [16]. Recently, viral titres during infection were correlated with the production of prostaglandin E_2_, which is known to be vasoactive [17].

Considering that dengue is an acute and short-termed disease, and IFN-α,β and TNF-α are molecules that induce antiviral defence mechanisms [11], it is conceivable that DENV replication is greatly inhibited in mononuclear phagocytes, although inflammatory reactions may be activated as well. It is well known that NO is produced by IFN-γ activation of monocytes [18] and has antiviral activities against Herpes Simplex Viruses, Epstein Bar Virus and some Coxsackie and Tick-borne encephalitis viruses [16]. NO levels were found to be increased in patients with dengue [19] or in vitro infected Kupffer cells [20], however the production has not been detected in infected monocytes in culture [21].

The aim of this research is to investigate iNOS monocyte induction during acute DENV infection in patients and after *in vitro *infection by DENV-1. We demonstrated that monocytes isolated from several patients became activated and express iNOS, which leads to the production of NO by the cell. DENV-1 was susceptible to a NO donor treatment and, in addition, virus was detected at higher rates in infected cells after iNOS inhibition, indicating that NO might play a substantial role in controlling DENV-1 infection of monocytes in culture and in vivo during natural infection.

## Methods

### Patients and laboratory diagnosis

Blood samples were obtained from DENV infected patients in April 2000 in Foz do Iguaçu, PR, and from February to April 2001 and January to March 2002 in Niterói, RJ, Brazil. Patients were diagnosed based on clinical grounds as Dengue Fever (fever, headache, retro-orbital pain, mialgias, arthralgias, rash and prostration); some had hemorrhagic manifestations, platelet counts under 100,000/mm^3^, hypotension receiving parenteral hydration and hospitalisation. The diagnosis of DENV infection was confirmed by anti-dengue enzyme-linked immunosorbent assay (ELISA)-IgM, or virus isolation. Informed written consent, approved by Fundação Oswaldo Cruz Ethical Committee under Nr#111/00, was obtained from all dengue patients prior to blood draw.

### Cell cultures

*Aedes albopictus *C6/36 cell clone was grown as monolayers at 34°C on Leibovitz medium (L-15) supplemented with 200 mM glutamine, 1% non-essential amino acids solution, 19% tryptose phosphate broth, 100 U/ml penicillin, 10 μg/ml streptomycin and 5% foetal calf serum (FCS).

### Preparation of virus stock and virus titration

DENV serotype 1, strain 16007 was provided by Dr. SB Halstead (Naval Medical Research Center, USA). Virus was titrated by serial dilution cultures in microtiter plates and detected by immunofluorescence as previously described [22]. Virus titre was calculated as 50 percent tissue culture infectious dose or TCID_50_/ml [23,24]. Inactivated virus was prepared by incubating the inoculum for 24 hours at 37°C and treating it with UV light (60 Hz, with distance of 20 cm) for 1 hour. Virus stock used was at a concentration of 3.18 × 10^7 ^TCID_50_/ml.

### Preparation of human peripheral blood mononuclear leukocytes (PBMLs)

Mononuclear leukocytes were obtained from heparinised venous blood originating from either DENV-infected patients or dengue seronegative adult donors. Cells were isolated through density gradient centrifugation (350 g, 20 minutes in Ficoll-Paque Plus Amersham Biosciences Corp, Piscataway, USA). Cells were suspended in RPMI 1640 supplemented with 200 mM glutamine, 100 U/ml penicillin, 10 μg/ml streptomycin and 10% FCS and afterwards incubated at 37°C under humid atmosphere with 5%CO_2_. Cells isolated from patients with acute dengue were suspended in supplemented RPMI 1640 containing an additional 10% DMSO and 50% FCS and maintained in liquid nitrogen.

### Infection of adherent PBMLs and treatment with an iNOS inhibitor

Infections were performed on 24-well plates. Freshly isolated PBML suspended in RPMI 1640 medium and supplemented with 10% FCS were seeded at 2 × 10^6^cells/well. After an 18 hour-incubation, adherent cells were enriched by washing away unattached cells twice. Inoculum was diluted in 1 ml medium containing a multiplicity of infection approximately of 8 TCID_50_/adherent cell DENV-1. After a 2 hour-incubation for adsorption, 1 ml medium was added to achieve 10% FCS. After an 18 hour-incubation, all culture medium containing virus was removed and cultures were further incubated with fresh medium for up to 6 days. In some experiments the iNOS inhibitor, N^G^-methyl L-Arginine (N^G^MLA), was added at a final concentration of 400 μM. Wells were set in triplicates for each different parameter in culture. Cell viability was determined in culture by Trypan blue exclusion during 6 days. Duplicates of cell control, inactivated and infectious DENV were assayed. Viability ratios were calculated by dividing cell counts from each day by counts made just after virus adsorption.

### Infection of C6/36 cell line and treatment with a NO donor

Infections were performed on 24-well plates. C6/36 were seeded at 2 × 10^5 ^cells/well in medium with 5% FCS and allowed to form a monolayer for 24 h at 34°C. Cell supernatant was removed and adherent cells were washed twice with medium without FCS. Wells were filled with 1 ml fresh medium containing DENV-1 or only medium in a 1:10 dilution from initial inoculum containing a multiplicity of infection of 1.59 TCID_50 _/ cell for DENV-1. After a 90 minute-adsorption, 1.0 ml medium was added to achieve 2% FCS, containing sodium nitroprussiate (SNP), a NO donor, resulting in final concentrations of 10 or 100 μM [25]. Viruses were allowed to grow for two days in incubated cultures. Triplicate wells were set for each culture parameter. Viability of treated cells was confirmed by Trypan blue exclusion or propidium iodine uptake.

### Virus labelling of infected C6/36 cells, single and double labelling of infected adherent PBMLs for flow cytometry analysis

Cells were recovered by scratching with plastic microtip using cold medium and were set at 1 × 10^6^/microtube; they were centrifuged (350 g, 5 min) and washed once with 1 ml PBS pH 7.4 with 2% FCS and 0,01% NaN_3_. Surface labelling was performed with FITC-labelled antibodies to CD14 (DAKO, Denmark, 1:100 dilution) for 45 min directly on adherent viable PBMLs under ice bath. This was done to confirm that ~95% of the monocyte gated cells would be CD14+ on the infection day. Intracellular staining after infection was performed according to previously described [26] with slight modifications. Briefly, it required a fixation with 0.5 ml cold paraformaldehyde at 4% in PBS for 10 min and, after centrifugation, membrane permeabilization was carried out with 1 ml 0.1% saponine in PBS with FCS and NaN_3_. Monoclonal antibody Dengue Complex-reactive (Chemicon, USA, 1:200 dilution in PBS with saponine, FCS and NaN_3_) was added to cells for a 60-minute incubation. Cells were washed once with 1 ml PBS with FCS and NaN_3 _and further incubated with anti-mouse IgG labelled with PE or FITC (DAKO, USA, 1:50 dilution in PBS with saponine, FCS and NaN_3_) for 30 minutes. After washing, infected adherent PBMLs were further incubated with FITC-labelled antibody to iNOS (B&D Transduction Laboratories, USA, 1:100 dilution) for 45 minutes. Alternatively, for double staining CD14 and DENV, cells were firstly labelled with PE-labelled antibodies to CD14 (DAKO at dilution 1:100) for 30 minutes 4°C, washed, fixed with 0,5 ml cold 2% paraformaldehyde for 10 minutes and, after centrifugation, membrane permeabilization was carried out with 1 ml 0.1% saponine PBS with FCS and NaN_3 _and further labelled with Alexa 654 – labelled monoclonal antibody to Dengue for 30 minutes. Matching isotype antibodies were used for DENV and iNOS labelings. Finally cells were washed twice, ressuspended in 1% paraformaldehyde and kept at 4°C up to 3 days until acquisition by flow cytometry. Cells were acquired (10,000 events for cell lines and 5,000 for gated monocytes) on a FACS^® ^Calibur flow cytometer (Beckon & Dickinson, USA) and analysed using FlowJo Software (TreeStar Inc., CA, USA). Isotype-matched antibodies were used as a negative control for all stainings.

### Statistical analyses

Two-way Student's *t *test was performed using GraphPad Prism version 4.02 for Windows, GraphPad Software (San Diego, CA, USA, ) in order to determine the significance of differences in percentages of virus labelling found in cells infected under various conditions. Altered parameters were considered significant when P < 0.05.

Labelled cell rates in healthy donors were firstly tested for normality using the Prism program (values passed the normality test of Kolmogorow-Smirnov) and then cell patient frequencies were tested for sample positivity by Student's *t-*Distribution (*t_n-1 = 10, α = 0.025 _*= 2.228 or *t_n-1 = 9, α = 0.025 _*= 2.262) calculating a referential limit value for negativity, according to the following formula: *Average of values from control samples + [Standard Deviation of values from control samples X t_(n-1;α = 0.025)_]*. Determinations above referential limit values were considered positive.

## Results

### Characterization of target cells for DENV and iNOS expression in peripheral blood mononuclear leukocytes (PBMLs) originating from patients with Dengue Fever

**Figure 1:**
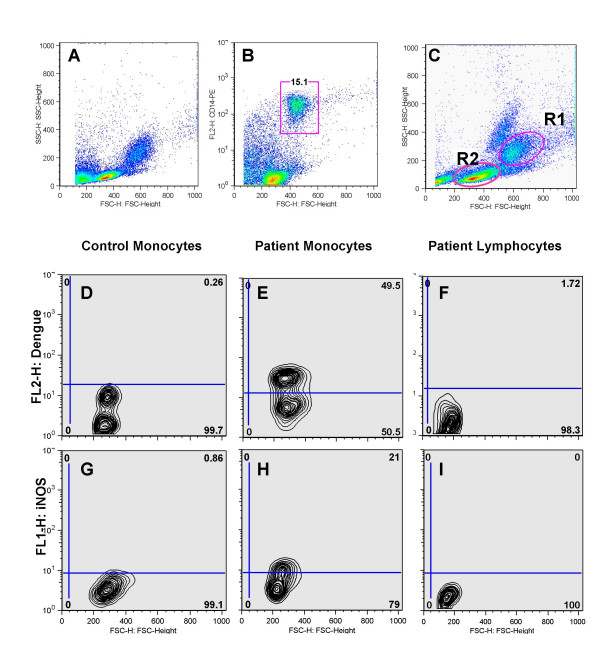
**Dot and contour plots showing DENV-Ag and iNOS monocyte and lymphocyte expression in dengue patients. **Human PBMLs a from healthy individual (Figures 1A,1D,1G) were used as control. Cells from a 4-day dengue infected patient (Figures 1B,1C,1E,1F,1H,1I) were labelled with anti-CD14-PE and CD14+ gated cells – R1 (Figure 1B) were considered as logical gate or *monocyte gate *and R2 is as the *lymphocyte gate *in the FCS *vs*. SSC dot plot (Figure 1C). R1 and R2 were used for further analysis during this work. Alternatively, human PBMLs were labelled either with an antibody to DENV Ags and anti-mouse IgG-PE (Figures 1D-1F) or with anti-iNOS-FITC (Figures 1G-1I) and analysed by FACS in either the R1, monocyte gate (Figures 1D-1F) or in the R2, lymphocyte gate (Figures 1F-1I). *x*-axis represent mean of cell population size (FCS) and *y*-axis represent cell granularity (SSC) or fluorescence intensity.

Previous reports demonstrated human monocytes as targets for DENV in cultures [1,27]. Therefore, monocyte and lymphocyte subsets present in PBMLs originated from patients with acute Dengue Fever were analysed by flow cytometry (FACS). Figure 1 displays the cell size and granularity profiles of PBMLs from a healthy donor (Figure 1A) and a dengue patient (Figure 1C). CD14+ gated cells were selected (Figure 1B) and plotted as logical monocyte gate in the FSCxSSC dot plot (R1 in Figure 1C). Monocyte (R1) and lymphocyte (R2) gates selected were used for further studies. Approximately 95% cells of the R1 are CD14+ cells.

**Figure 2:**
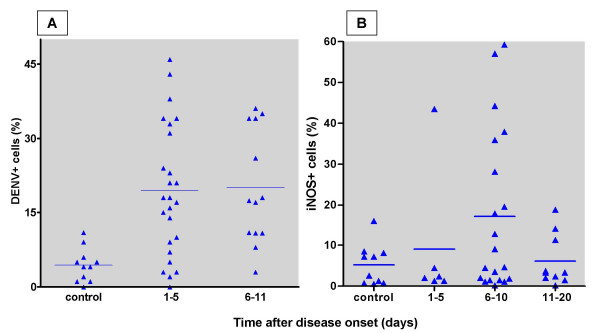
**Frequencies of DENVAg+ and iNOS+ monocytes in dengue patients.** Human PBMLs from dengue individuals were labelled with an antibody to DENV-Ag and an anti-mouse IgG-PE or with an antibody to iNOS FITC-labelled and data obtained by FACS in the monocyte gate. Percentages of DENV-Ag+ infected cells were determined in 11 healthy individuals and 35 with dengue (Figure 2A; 2 patients were measured in duplicate samples at different time points). Percentages of iNOS+ infected cells were determined in 10 healthy individuals and 22 with dengue (Figure 2B; 14 patients were measured in duplicate samples at different time points). Horizontal lines represent percentage averages of labelled cells from the population of individuals in each of the discrete categories.

The monocyte and lymphocyte profile by FACS after DENV and iNOS immune-labelling were studied. Representative dot plots of DENV Antigens (Ag) and iNOS positive detection in monocytes from dengue patients (Figures 1E and 1H, respectively) are compared to monocytes from healthy individuals (Figures 1D and 1G) and lymphocytes from patients (Figures 1F and 1I). Patient monocytes presented DENV-Ag during the first eleven days of disease onset (Figures 1E and 2A) compared to control monocytes (Figures 1D and 2A). Patient cells collected at later stages of disease did not display detectable viral antigen (data not shown). Among the 35 dengue patients 25 expressed DENV-Ag above the referential limit value for negativity in the Student's *t-*Distribution and no difference was detected between early (1–5 days) and late (6–11 days) infection. Among 22 patients tested for iNOS expression (Figures 1H and 2B), 10 were positive as compared to controls (Figures 1G and 2B). Cells from the lymphocyte gate do express neither DENV-Ag (0.80 ± 0.77%) nor iNOS (3.7 ± 2.57%) (Figures 1F and 1I, respectively). The highest incidence of iNOS activation was from 6 to 10 days of disease (8 out of 20 patients). Among 11 patients with platelet counts<100,000/mm^3^, six had iNOS+ cells above the referential limit value for negativity. Seven of these patients also had hypotension, were hospitalised and submitted to parenteral rehydration. Among 11 patients with normal platelet levels merely 4 had significantly elevated iNOS^+ ^cell ratios. INOS expression, present in a significant frequency among dengue patients, could not be associated with severity in this sampling.

### Characterization of DENV target cells, DENV-Ag detection and iNOS expression in monocytes after in vitro infection

Adherent human PBMLs were incubated for 3–6 days with infectious and inactivated DENV and evaluated for presence of DENV-Ag and iNOS expression. Cell viability was determined daily by Trypan blue exclusion. After 3 days of culture, cells incubated with the inactivated virus remained 42 ± 7% viable and with infectious virus 42 ± 23% viable as compared to the day 0. On day 6 37 ± 2% cells were viable for inactivated and 44 ± 3% for infectious virus, showing no differences between the two virus incubations. Control cell rates of viability were higher, 58 ± 8% at day 3 and 69 ± 19% at day 6. Although infectious viruses may not be crucial in increasing monocyte mortality, viral antigens alter cell viability.

Kinetics of virus detection in mononuclear phagocytes varies according to virus strain and multiplicity of infection (MOI) used (Cologna *et al*., 2003). At the MOI used here for DENV-1 strain 16007 (8 TCID_50_/adherent cell) preliminary results displayed low rates of infection on the first day (in average 17 ± %), increasing at day 2 (average 31%). No iNOS labelling was detected on these first days of infection. Therefore, experiments were performed using infections from 3 to 6 days.

**Figure 3:**
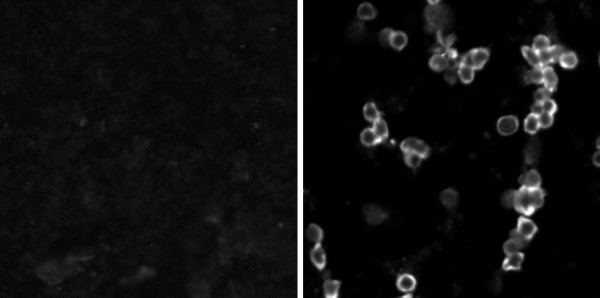
**Confocal microscopy of DENV-Ag expression after *in vitro *infection of monocyte-rich cultures. **Adherent human PBMLs were incubated for three days either with cell culture medium (Figure 3A, 189.5 μm/field), or with infectious DENV (Figure 3B, 125 μm/field). Cells were labelled with antibody to DENV-Ag and anti-mouse IgG-FITC.

**Figure 4:**
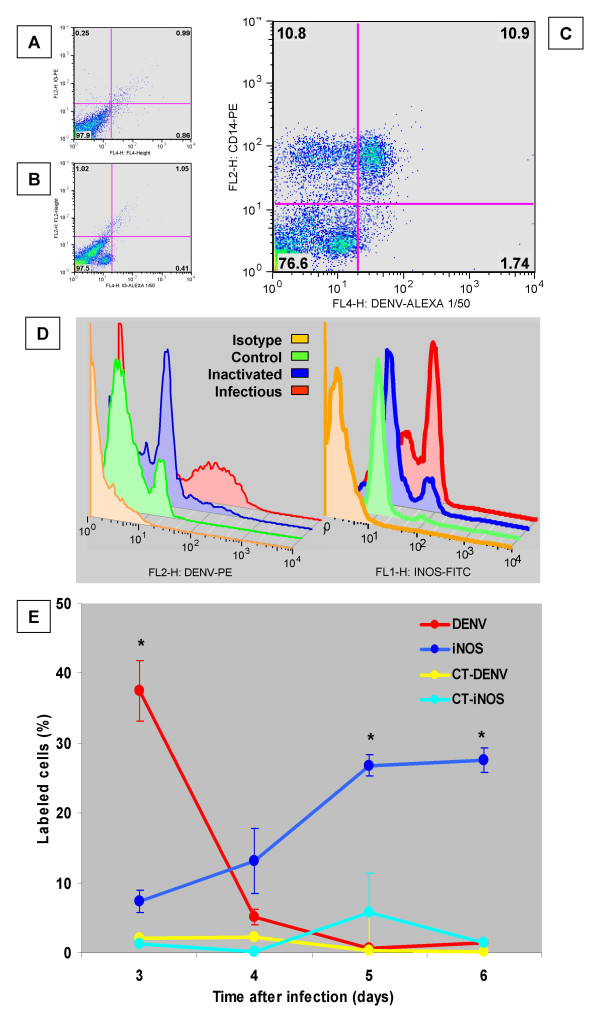
**DENV-Ag and iNOS expression after *in vitro *Dengue infection of monocytes. **Adherent human PBMLs were incubated for three days with infected DENV (Figures 4A, 4B and 4C). Cells were labelled either with isotype matched antibodies (Figures 6A and 6B) or antibodies to CD14-PE (*y*-axis) and DENV-Complex-Alexa-654 (*x*-axis) and were analysed by FACS (Figure 4C, *ungated*). Cells incubated for three to six days with cell culture medium, inactivated DENV or infectious DENV were labelled with antibody to DENV-Ag and anti-mouse IgG-PE and/or anti-iNOS-FITC and analysed by FACS. Isotype matched antibodies were used for both labelings (Figure 4D, 3 days for DENV-Ag and 6 days for iNOS labelling). *x*-axis represent mean of fluorescence intensity and *y*-axis represent percentage of maximum cell number counts. Figure 4E shows a kinetic curve comparing DENV-Ag and iNOS expression on cells from the monocyte gate. Each point represent average ± SEM percentages from samples set in triplicates. * *P < 0.05 *and in Student's T-test showing difference in DENV-Ag and iNOS expression compared to uninfected controls (CT). Data are obtained from one representative experiment out of three performed with different cell donors.

Figure 3B shows an image obtained under confocal microscopy in a field with a high frequency of infected adherent mononuclear leukocytes compared to the absence of fluorescent cells in control cells (Figure 3A). DENV-Ag^+ ^cells were detected by flow cytometry among CD14^+ ^cells, although very small rates of CD14^- ^cells were also labelled for DENV-Ag (Figures 4A and 4B are labelling controls for Figure 4C). For further evaluations during *in vitro *infection, we studied the monocyte population, using the CD14^+ ^logical gate, which presented the same profile as the R1 shown in Figure 1B).

In Figure 4D we observed that infected monocytes displayed specific DENV-Ag expression when compared to monocyte incubation with inactivated virus and an isotype-matched antibody, suggesting virus replication during infection. INOS was specifically expressed in monocytes infected with DENV. Mock-infected monocytes by inactivated DENV did not yield viral antigens during culture. The inactivated virus consistently induced low rates of INOS+ monocytes, however differences were statistically insignificant when compared to controls. The infectious virus in all experiments resulted in significantly higher rates of INOS^+ ^monocytes than in control cultures. Infectious virus, if not crucial, certainly play an important role in inducing iNOS. Control cells displayed only low levels of background labelling for both DENV-Ag and iNOS.

During *in vitro *infection DENV-Ag were markedly expressed in monocytes and detected on day 3 either by confocal microscopy (Figure 3B) or by flow cytometry (Figure 4). However, from day 4 to 6 Ags detection disappeared rapidly (Figure 4E) with insignificant rates of labelled cells (Student's T-test). INOS is apparently detected in cells already at day 3, but it reaches significantly higher frequencies of positive cells at days 5 and 6 (Figure 4E).

### DENV-Ag detection in infected cells after treatment with an iNOS inhibitor – N^G^MLA or with a NO donor – SNP

**Figure 5:**
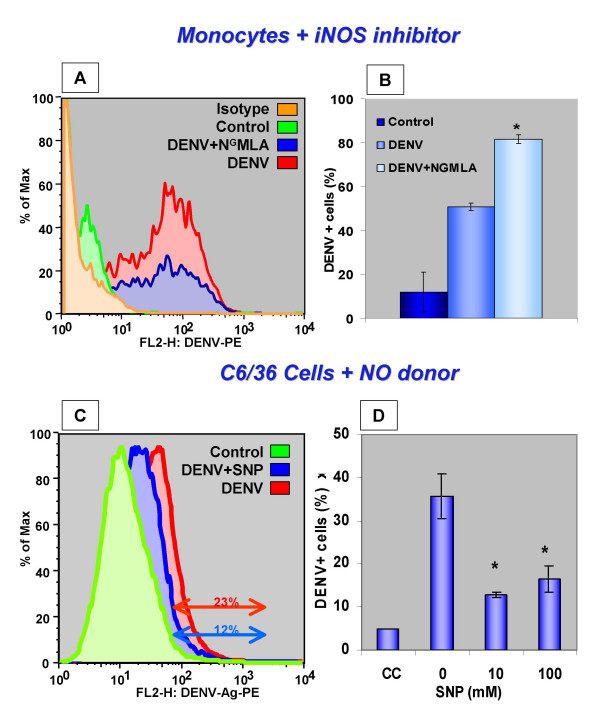
**Effect of an iNOS inhibitor and of a NO donor on DENV-Ag expression after cell infection. **Figures 5A and 5B – Effect of iNOS inhibitor – NGMLA on monocyte infection. Adherent PBMLs were incubated with either Mock C6/36 cell supernatant or DENV inoculum for 4 days in presence or absence of 400 μM NGMLA. Figure 4A shows histogram with profile of infected monocytes after NGMLA treatment and labelling controls. Figure 4B shows percentages of DENV-Ag+ in gated monocytes obtained by flow cytometry. Figures 5C and 5D -. Effect of NO donor – SNP, on C6/36 cell infection. C6/36 cells were incubated with DENV for 2 days in absence or presence of 10 or 100 μM SNP. Figure 4C shows a histogram with profiles of infected C6/36 cells obtained by FACS from a culture treated with 10 μM SNP or controls. The histogram gates have been made where ≥99% than cells in the cell control are excluded. Figure 4Dshows percentages of DENV-Ag+ cells calculated from ungated cells after labelling with antibody to DENV-Complex and anti-mouse IgG-PE. In histograms *x*-axis represent mean of fluorescence intensity and *y*-axis represent percentage of maximum cell number counts. Cells were labelled with antibody to DENV-Complex followed by anti-mouse IgG-PE. Average ± SEM are represented from samples set in triplicates. * *P < 0.05 *in Student's T-test showing difference in DENV infection after treatment. Data are obtained from one representative experiment out of two for each cell type using two different monocyte donors.

Nitric oxide antiviral activity induced by viruses in mononuclear phagocytes may be inhibited by N-monomethyl-L-arginine acetate (N^G^MLA) [28]. Monocyte-enriched cultures were treated with N^G^MLA and simultaneously infected with DENV. DENV-Ag^+ ^cells were present in significantly higher frequency among infected cells in treated cultures after 4 days, as compared to infected and untreated cultures (Figure 5A, 5B). Figure 5A presents a flow cytometry histogram with an increased DENV-Ag^+ ^cell population in presence of N^G^MLA treatment as compared to untreated infected cultures, with significantly higher percentages of labelled cells (Figure 5B). Thus, the inhibition of iNOS facilitates DENV-1 replication, suggesting that NO production might be involved in the control of virus replication.

Dengue virus has limited rates of infection in monocytes and after a few days infection is controlled accompanied by the induction of several factors that may act as inhibitors of viral replication [21,29]. Since C6/36 cells are extremely susceptible to DENV infection and allow intense virus replication, these mosquito cells should be suitable to test the effect of a potential antiviral molecule. Therefore, C6/36 cultures were infected with DENV and simultaneously treated with sodium nitroprussiate (SNP), a known NO donor, which was added at concentrations of 10 or 100 μM. After 2 days of infection and treatment, DENV-Ag+ cells were detected using FACS. An uninfected cell control was used in order to detect unspecific labelling. SNP induced a significant inhibition of DENV-Ag expression in infected C/36 cells in both tested concentrations, when percentages of positive cells were compared to those in untreated infected cells (analysis by Student's T test; Figures 5C and 5D). Therefore, inhibition of DENV-1 replication by NO production is evident during the present experiments.

## Discussion

DENV was detected for the first time by flow cytometry in peripheral monocytes of infected patients. Earlier reports demonstrated viral particles in monocytes, lymphocytes or endothelial cells from tissues and blood clot of patients [9,30]. Infectious DENV [3] or PCR detection of nucleic acid [7] in PBMLs were even at higher incidence than in serum. We did not detect viral antigens in lymphocytes despite others having done so [9,30]. This may be due to the viral strain used or alternatively the genetic constitution of the human PBML donors. Monocytes are important targets for DENV either *in vitro *[31] or *in vivo *[8]. Supporting our previous report [26], we present here that DENV likely replicates in monocytes since detected antigens increase with time and no antigen is detected when inactivated virus is used. This indicates that viruses probably replicate for a short period and, during cell infection, mechanisms are activated that are able to control further replication.

Interferon (IFN)-mediated antiviral action is still considered to be among the most important intracellular mechanisms against viruses, however recently a mechanism in which viral proteins might be involved in blocking IFN signalling during dengue virus infections has been described [32] supporting the conjecture that other complementary antiviral activities may be acting during dengue infection as well [33].

We have exhibited here that DENV can be detected in peripheral monocytes of patients with acute Dengue Fever, and that these cells are activated as demonstrated by the expression of iNOS studied *ex vivo*. Equivalent data was obtained with in vitro infected monocytes from healthy individuals, and virus presence can be modulated by inhibiting iNOS or generating NO in cell culture, indicating that this molecule is likely to have a role in controlling DENV-1 genome expression within monocytes.

During acute Dengue Fever, iNOS expression in monocytes differed among patients probably due to individual variations. Inducible NOS is encoded by the NOS2A gene, haplotype 2 is associated with self-limiting hepatitis C infections [34] and NO production was associated with less severe forms of dengue [19].

Viral replication inhibition by NO has been considered with even increasing importance. The ability of NO to inhibit virus replication was first described in 1993 [33,35,36] and thereafter many other studies have been developed. While some viruses were reported to be susceptible to NO action, others were resistant [35]. The mechanisms by which NO induces viral modulation are diverse, such as inactivation of viral cysteine protease by NO-dependent S-nitrosylation [36] or inhibition of Epstein-Barr virus reactivation. The inhibition of virus replication by NO was found to be mediated by IFN-γ [33,37] and dependent upon the induction of the signal transducer and activator of transcription (STAT) STAT1 phosphorylation [38]. Antiviral NO-mediated effect may also be activated by IFN-α, since it has been observed that mononuclear leukocytes activated *in vitro *by IFN-α or from Hepatitis C virus infected patients undergoing IFN-α therapy produce NO [39]. Some flaviviruses had their NO susceptibility tested and an effective inhibitory mechanism was determined for Japanese encephalitis virus [40], but studies on Tick-borne encephalitis virus (TBE-V) revealed no antiviral effects by NO [35]. The fact that dengue patients may have monocytes activated by circulating IFN-γ [12-14] is consistent with induction of iNOS during acute disease as reported here for both *in vivo *and *in vitro *infection.

NO may play a role in severity of viral hemorrhagic fevers(Sanchez *et al*., 2004) probably when produced in high concentrations, by affecting vascular tone and contributing to virus-induced shock. As previously mentioned, NO was detected in sera from patients with dengue [19] and correlated with the less severe form of the disease. Hence, NO may be exerting both protective and pathological action, depending upon the concentration produced [41]. Since our panel consisted of few patients, a correlation was not possible; iNOS+ cell rates were increased in some patients from both groups presenting mild or severe disease. Espina *et al*. [21] were not able to detect NO production by monocytes in culture. This apparent contradiction with our data of induced NOS may be explained by the use of a diferent detection technique: Griess reagent detects nitrite in cell culture supernatant which may be below method sensitivity since the density of monocytes in culture is not high. We did not succeed in detecting NO by this method in human monocyte cultures, and we only detected NO in murine macrophages after proper stimulation at high cell culture density [20]. Moreover, detection of NO produced in vitro by infected Kupffer cells [20] was carried out by a fluorimetric assay, much more sensitive than the Griess Reaction.

Only few reports show human mononuclear cells from healthy individuals generating NO production following treatment with cytokines, pathogens, and/or pathogen-derived products [39,42]. Until a few years ago expression of iNOS in humans was questioned [43] or difficult to detect [20,44]. It may be possible that iNOS induction and expression dependent on synergistic signals to macrophage/monocyte [45,46], and cytokines and virus products may be acting as different stimuli.

Replicating dengue viruses may be necessary for optimal iNOS induction, since rates of iNOS+ cells were much higher than when the inactivated virus was used. However, Kupffer cells infected with DENV induced NO and iNOS, in spite of the absence of viral progeny. Non-living products, such as peptides [47], originating from either other viruses [39,48] or parasites [49,50] can stimulate iNOS in macrophages/ monocytes and induce NO production.

Our data demonstrate that, when experimentally infecting their targets (e.g., monocytes), DENV-1 is able to induce a progressive expression of the enzyme iNOS, indicating that NO might be produced during *in vitro *infection as it is produced *in vivo*. At 6 days of infection, the iNOS detection is increased as compared to that of 3 days and is coincident with the DENV decrease, favouring the hypothesis that iNOS^+ ^cells were promoting an inhibitory effect on viruses either directly by NO production or by other virus-induced mechanisms during this monocyte activation, such as IFN-α production [39]. Alternatively cells interacting with viral antigens are probably becoming activated and dying either by cell lysis or by virus induced apoptosis [21]. We also observed that surviving cells remained in culture for more than10 days : either viruses are eliminated or cells become refractory by interferon-related mechanisms, in contrast with what is observed, e.g., for mosquito C6/36 cells, which are mostly destroyed by the virus. Here we observed that there is an enhancement of DENV-1 Ags detection, when infected monocytes were cultured with the iNOS inhibitor N^G^MLA, indicating that DENV-1 is very likely susceptible to NO inhibitory activity. This result is confirmed by the observation that DENV-1 detection is strongly inhibited in C6/36 cultures by the presence of the NO donor, SNP. These findings are similar and supported by observations in other viral models [51].

With regard to DENV replication, further studies are warranted in order to elucidate which pathways are involved in NO-mediated inhibition of DENV-1. Despite C6/36 remain viable by Trypan blue or propide iodine exclusions, it has to be considered that the NO action in virus targeted cells may involve apoptosis. Furthermore, it is not known whether or not other strains and serotypes will activate iNOS and be sensitive to NO action. There may be relevance in studying various strains from different serotypes and evaluate if susceptibility and resistance are strain and/or serotype specific.

## Conclusion

The data presented here displays for the first time the presence of iNOS in monocytes from patients with acute dengue fever. Despite the fact that, in excess, NO may provide a deleterious effect on vascular permeability, evidence exhibited here favour a role for NO in the control of *in vitro *dengue infection within monocytes. This may hold true for natural human infection as well.

## Competing interests

The author(s) declare that they have no competing interests.

## Authors' contributions

PCFNS and ELA participated equally in the study design, in the experimental work and in the preparation of the manuscript.

PCFNS carried out all the *in vitro *infections and assays and flow cytometry analysis.

ELA carried out all the work with patient blood collecting, storing cells and plasma and performing flow cytometry assays and analysis.

SMOZ established the connection with the Health Units in Niterói, examined the patients and registered the clinical data.

RVS examined the patients and registered the clinical data.

SRNIR helped with patient blood collecting, storing cells and plasma.

DISC helped to perform flow cytometry assays.

RMRN was responsible for the confirmatory diagnosis of dengue in Rio de Janeiro.

CFK oversaw the study and participated in its design and coordination, performed flow cytometry and statistical analysis and prepared the initial and revised drafts of the manuscript and figures.

## Pre-publication history

The pre-publication history for this paper can be accessed here:


